# Subthalamic deep brain stimulation induces finely-tuned gamma oscillations in the absence of levodopa

**DOI:** 10.1016/j.nbd.2021.105287

**Published:** 2021-05

**Authors:** C. Wiest, G. Tinkhauser, A. Pogosyan, S. He, F. Baig, F. Morgante, A. Mostofi, E.A. Pereira, H. Tan, P. Brown, F. Torrecillos

**Affiliations:** a Medical Research Council Brain Network Dynamics Unit, University of Oxford, Oxford, UK; b Nuffield Department of Clinical Neurosciences, John Radcliffe Hospital, University of Oxford, Oxford, UK; c Department of Neurology, Bern University Hospital and University of Bern, Bern, Switzerland; d Neurosciences Research Centre, Molecular and Clinical Sciences Institute, St. George's, University of London, London, UK

**Keywords:** Parkinson's disease, Local field potentials, Finely-tuned gamma, Adaptive deep brain stimulation, Feedback markers

## Abstract

Finely-tuned gamma (FTG) oscillations can be recorded from cortex or the subthalamic nucleus (STN) in patients with Parkinson's disease (PD) on dopaminergic medication, and have been associated with dyskinesias. When recorded during deep brain stimulation (DBS) on medication the FTG is entrained to half the stimulation frequency. We investigated whether these characteristics are shared off medication by recording local field potentials (LFP) from the STN from externalised DBS leads in 14 PD patients after overnight withdrawal of medication. FTG was induced *de-novo* by DBS in the absence of dyskinesias in a third of our cohort. The FTG could outlast stimulation or arise only after DBS ceased. FTG frequencies decreased during and across consecutive DBS blocks, but did not shift with changing stimulation frequency off medication. Together with the sustained after-effects this argues against simple entrainment by DBS in the off medication state. We also found significant coherence between STN-LFP and electroencephalogram (EEG) signals at FTG frequencies. We conclude that FTG is a network phenomenon that behaves differently in the off medication state, when it is neither associated with dyskinesias nor susceptible to entrainment.

## Highlights

• DBS induces *de-novo* finely-tuned gamma (FTG) oscillations in the subthalamic LFP
• Induced FTG appears off levodopa and without dyskinesias
• FTG frequencies change within and across consecutive stimulation periods
• STN-LFP and cortical activities are coherent at FTG frequencies

## 1 Introduction

A finely-tuned gamma (FTG), occupying a narrow frequency band within the 60 and 90 Hz range, has been reported in the subthalamic nucleus, globus pallidus, thalamus and cortex in patients with various neurological disorders (Alegre et al., 2005; Alonso-Frech et al., 2006; Androulidakis et al., 2007; Brown et al., 2001; Foffani et al., 2005; Fogelson et al., 2006; Kempf et al., 2009; Litvak et al., 2012; Miocinovic et al., 2018; Swann et al., 2016). The FTG appears as a network oscillation in so far as it is coherent between sites in the cortico-basal ganglia-thalamic loop (Brown et al., 2001; Fogelson et al., 2006; Kempf et al., 2009; Litvak et al., 2012). In unexaggerated form it may be a primarily physiologic phenomenon related to arousal and voluntary movement, and its sharpness distinguishes it from canonical broadband gamma reactivity in the cortico-basal ganglia system (Jenkinson et al., 2013; Litvak et al., 2012). In patients with Parkinson's disease (PD), FTG appears following medication with levodopa and is further increased during voluntary movements (Brown et al., 2001; Alonso-Frech et al., 2006; Androulidakis et al., 2007), observations that have led it to be considered a prokinetic rhythm (Androulidakis et al., 2007; Brown, 2003; Cassidy et al., 2002; Litvak et al., 2012). Hence, the extent of amplification of FTG during voluntary movement correlates positively with movement velocity in PD (Lofredi et al., 2018), although a broader gamma increase has also been linked to successful stopping of movements (Fischer et al., 2017). Moreover, when excessively exaggerated, FTG in the basal ganglia thalamo-cortical loop may contribute to involuntary movements, such as levodopa-induced dyskinesias in PD (Swann et al., 2016), or dystonic posturing in isolated dystonia (Miocinovic et al., 2018). The link to levodopa-induced dyskinesias has also been noted in animal models of Parkinsonism (Güttler et al., 2020).

The generally prokinetic effect of FTG has led to speculation that it may mediate some of the effects of deep brain stimulation (DBS). Thus stimulating the subthalamic nucleus (STN) with individually detected FTG-frequencies can produce similar clinical benefit as stimulating with frequencies above 100 Hz (Tsang et al., 2012). Indeed, cortical FTG may shift in frequency to half that of DBS during stimulation of the STN, signalling possible entrainment by stimulation when dyskinesias appear on medication (Swann et al., 2016). However, what happens to FTG during DBS when off medication or when dyskinesias are absent? Is it still affected by DBS, and if so in what way? Technical issues mean that, hitherto, it has been easiest to record LFP activity in the STN immediately after DBS to this target, and one study has reported that 60–90 Hz activity in the STN is not temporarily increased following STN-DBS performed off medication (Foffani et al., 2006). However, this study averaged gamma activity over patients with PD and as FTG is sharply tuned and inconsistent between patients, modulation may have been missed (Foffani et al., 2005). Another study managed to record FTG during DBS using high-density EEG and demonstrated a correlation between the power at FTG frequencies in both cortical regions and STN, which was derived using a beamformer algorithm, and local power at the stimulation frequency (Muthuraman et al., 2020). This occurred without a shift in the frequency of the presumed FTG activity, although the two stimulation frequencies used had subharmonics that were already close to the frequency of FTG. Moreover, this study reported co-modulation and did not directly demonstrate endogenous FTG. Here we explore whether STN-DBS off medication has an effect on local FTG activity allowing for the fact that this activity may only be recorded in a subset of patients (“high gamma” activity in López-Azcárate et al., 2010). We address two major questions; is FTG in the STN promoted by DBS, appearing *de-novo* with stimulation, and if so is the new activity entrained by DBS?

## 2 Materials and methods

### 2.1 Consent, regulatory approvals and patient selection

This protocol was approved by the Health Research Authority UK and the National Research Ethics Service local Research Ethics Committee (IRAS: 46576). Patients were recruited at St. George's University Hospital NHS Foundation Trust, London. Written informed consent was obtained in line with the Declaration of the Principles of Helsinki. Fourteen patients with PD (1 female) undergoing bilateral STN-DBS surgery were included in the study. Their average age at the time of recording was 57.2 ± 1.6 years (mean ± SEM) with average disease duration of 10.1 ± 1.2 years. Three patients were recorded bilaterally, giving a total of 17 STNs included in the study. Clinical details are summarised in Table 1. Data from patients 1 to 9 and patients 11 and 12 were previously published (Wiest et al., 2020).

**Table 1:** Patients clinical and recording details. Pre-OP: pre-operative; Post-OP: post-operative; UPDRS-III: Unified Parkinson's disease Rating Scale (subunit III); FTG: finely-tuned gamma; Boston: Boston Scientific; Medt: Medtronic; STN (SP): superior-posterior part of the subthalamic nucleus; FOG: freezing of gait; STN/cZI: subthalamic nucleus/caudal Zona incerta; LID: levodopa-induced dyskinesia; RDRS: Rush Dyskinesia Rating Scale; DID: DBS-induced dyskinesia; n.a.: not applicable.

Patient	Gender (m/f)	Age (yr)	Disease Duration (yr)	Pre-OP UPDRS-III OFF	Pre-OP UPDRS-III ON	Pre-dominant symptoms	DBS lead	Time Recording (d)	STN tested (L/R)	Location Stim. Contact	Stim. Amp. (mA)	FTG present OFF meds ON DBS	LID contralat. to tested STN pre-OP (RDRS)	DID contralat. to tested STN post-OP
1	m	48	17	71	37	tigidity, gait freezing (FOG), tremor	Boston	4	R	STN (SP)	2	yes	yes (6)	no
2	m	54	7	38	24	tremor	Boston	5	R	STN (SP)	3.5	yes	no (0)	yes
3	m	51	5	27	13	bradykinesia, rigidity, motor fluctuations	Boston	4	R	STN/cZI	4	yes	no (0)	yes
4	m	64	13	52	21	FOG, motor fluctuations	Boston	4	R	STN (SP)	2	yes	no (0)	no
5	m	53	7	23	12	Tremor	Boston	4	R	STN (SP)	2	no	no (0)	no
								5	L	STN (SP)	3	yes	no (0)	no
6	m	61	16	50	30	FOG, motor fluctuations	Boston	3	L	STN (SP)	2	no	no (0)	no
7	m	56	16	51	19	Rigidity, bradykinesia	Medt	4	L	STN (SP)	4	no	no (0)	no
8	m	65	5	34	16	FOG, motor fluctuations	Boston	4 + 5	L	STN/cZI	3	no	yes (2)	no
								4	R	STN/cZI	2.5	no	yes (3)	yes
9	f	63	11	40	17	FOG, motor fluctuations	Boston	4	L	STN (SP)	2	no	yes (2)	no
10	m	65	15	77	27	Akinetic-rigid, tremor	Boston	5	L	STN (SP)	1.8	no	yes (1)	no
11	m	61	9	33	12	Rigth arm tremor, bradykinesia	Boston	4	L	STN/cZI	4	no	no (0)	no
12	m	48	8	45	34	Tremor, bradykinesia	Boston	5	L	STN/cZI	3	no	no (0)	no
13	m	59	6	48	14	Akinetic-rigid	Medt	5	R	STN (SP)	n.a.	no	yes (1)	no
14	m	53	7	18	1	Motor fluctuations, dyskinesia	Boston	4	L	STN/cZI	4	no	no (0)	no
								4	R	STN/cZI	2.5	no	yes (1)	no

### 2.2 Clinical characterisation

Study participants were evaluated by an interdisciplinary team of movement disorder neurologists and functional neurosurgeons and met the UK Parkinson's Disease Society Brain Bank Diagnostic Criteria for diagnosis of PD (Hughes et al., 1992). Baseline motor function in the on and off medication state was characterised pre-operatively using part III of the Unified Parkinson's disease Rating Scale motor subscale (UPDRS III). Patients were evaluated by a neuropsychologist to exclude significant cognitive impairment or untreated affective disorders. During the recordings, an experienced neurologist was present to screen for dyskinesia.

### 2.3 Surgery and lead localisation determination

The surgical target was STN. Two models of DBS leads were used; either a quadripolar subthalamic lead (model 3389, Medtronic Inc., Neurological Division, USA) with four 0.5 mm spaced contacts of 1.5 mm length with platinum‑iridium cylindrical surfaces or a directional subthalamic lead (model DB-2202, Boston Scientific, USA) with three segmented contacts on each of the middle two levels. These middle two levels sat above and below a concentric electrode contact. Electrodes were implanted using frame-based stereotaxy and MRI guidance. The subthalamic leads were connected to temporary extension leads and these externalised over the left temporal bone. Assessment of contact localisation was made through co-registration of immediate post-operative CT with pre-operative MRI by two experienced neurosurgeons (E.A.P and A.M.) specialising in DBS. Assessment was blinded to the electrophysiological data and made using Renishaw Neuroinspire v6 software. Localisation specifics are summarised in Table 1.

### 2.4 Stimulation and data recording

**Fig. 1:**
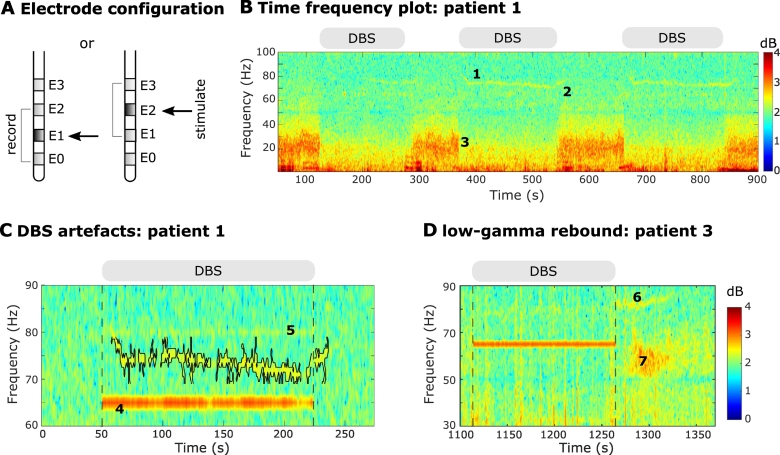
Stimulation settings and experimental paradigm. A. Schematic illustration of the electrode configuration. DBS was applied to one of the two middle contacts (E1 or E2) and LFPs were recorded bipolarly from the two adjacent contacts (E0 and E2 or E1 and E3). Bipolar contacts were recorded directly and not recovered off-line. B. Time-frequency spectrogram of first three DBS blocks in patient 1. During stimulation the finely-tuned gamma (FTG) arises (label 1) and then outlasts DBS (label 2) when it increases in frequency. In addition, beta suppression is evident during DBS (label 3). DBS blocks at 130 Hz are denoted by the grey bars at the top of the trace. Note that line noise artefacts were present throughout the recording at 50 Hz along with the DBS subharmonic at 65 Hz but are reduced by second-order IIR notch filters. C. DBS-induced narrowband artefacts at a subharmonic DBS frequency (65 Hz; label 4) or due to an aliasing phenomenon (80 Hz; label 5). Time-frequency spectrum shown for block 2 of patient 1with DBS at 130 Hz and before applying IIR notch filters (see Methods). The grey bar and vertical lines denote DBS onset and offset. The FTG feature is contoured (see Methods). Note that DBS-induced artefacts do not change in frequency throughout stimulation and cease immediately after DBS is stopped. D. Representative time-frequency spectrogram from patient 3 block 2. Apart from the FTG after DBS (label 6) we observe a low-gamma rebound (label 7) which appears after DBS is stopped with a slight delay, spans from 50 to 70 Hz and lasts for several seconds.

Recordings were made between 3 and 5 days postoperatively (Table 1), while electrode leads were still externalised and before implantation of the subcutaneous pulse generator. All patients performed the experiments after overnight withdrawal of antiparkinsonian medication (Table 1). In patients with directional leads, the three directional contacts of level 1 and 2 were joined to form two ‘ring contacts’ so that each lead afforded four monopolar ring contacts. The four contacts were numbered E0 to E3 with contact E0 being the most ventral and contact E3 being the most dorsal (see Fig. 1A). Stimulation was only tested at the two middle contacts (E1 and E2) to allow bipolar LFP recordings from the two adjacent contacts. A self-adhesive electrode (Pals, Nidd Valley Medical Cordon, UK) attached to the patients' back served as a reference for stimulation, which was delivered using a highly configurable neurostimulator (Slater et al., 2015). Stimuli comprised symmetric constant-current biphasic pulses (60 μs per phase, negative phase first). In each of the two middle contacts, stimulation was started at 0.5 mA and slowly increased in steps of 0.5 mA every 3 to 4 min until first a clear benefit in rigidity and/or bradykinesia was observed and second side effects such as paraesthesia became apparent. The stimulation contact and current associated with the most clinically effective stimulation without side effects was selected and maintained throughout the entire experiment (DBS currents listed in Table 1). This procedure was performed on the side contralateral to the most affected upper limb in 11 subjects and bilaterally in 3 subjects (Table 1). If not indicated differently in the results, a stimulation frequency of 130 Hz was used. STN-LFPs were recorded in bipolar mode from the two contacts on either side of the stimulation contact, for common reference removal ((Schiff, 2005) and see Fig. 1A). Limited concomitant EEG electrodes were placed at Fz, FCz, Cz, Oz, C3, C4, CP3 and CP4 according to the extended 10–20 EEG system and allowing at least a 3 cm distance from all incisions. EEG time series were recorded with a common average reference (that did not include LFP channels). All signals were amplified and sampled at 2048 Hz using a TMSi Porti (TMS International, Netherlands) and custom-written software developed using the C programming language. The ground electrode was placed on the forearm.

### 2.5 Experimental design

During the experiments, patients were comfortably seated in an armchair. Note that recordings were performed using different experimental protocols (some already published, see Wiest et al., 2020). Only a few patients were tested at different stimulation frequencies (patients 3, 4, 8 and 9) and durations (patients 4, 5, 6 and 9). However, all recordings started with two minutes of rest recording without stimulation (baseline) followed by blocks of continuous 130 Hz DBS. Stimulation blocks lasted for 163.1 ± 11.1 s (mean ± SEM) in up to four consecutive blocks separated by resting periods of on average 110.2 ± 9.9 s. Additional blocks with different DBS frequencies were tested in two of the five subjects showing the FTG (see Table 2). In patient 3, two blocks at 70 Hz DBS (150.5 ± 1.4 s) were recorded followed by two blocks at 180 Hz (153.5 ± 4.0 s), with resting periods of on average 118.9 ± 2.7 s between them. In patient 4, one DBS block at 180 Hz for 24.0 s and another block at 190 Hz for 24.1 s separated by a series of short DBS bursts at the same frequency (25 bursts of on average 115.2 pulses at 180 Hz and 15 bursts of on average 143.5 pulses at 190 Hz; Fig. 5B) were recorded. Also in patient 4, four short duration DBS blocks (13.1 ± 0.2 s) were recorded at 70 Hz, 130 Hz and 190 Hz each (see Table 2), each separated by resting periods of on average 149.0 ± 3.9 s.

**Table 2:** Details of the finely-tuned gamma. Electroencephalogram (EEG) contacts are labelled according to the extended 10–20 EEG system at Fz, FCz, Cz, Oz, C3, C4, CP3 and CP4. FTG duration, amplitude and frequency were extracted using a cluster based approach (see Methods). Freq.: frequency; R: right; L: left; n.a.: not applicable.

Patient	number of long DBS blocks	DBS Freq. (Hz)	FTG during DBS (at 130 Hz)	FTG duration during DBS (% of block) (mean & range)	FTG Freq. during DBS (Hz) (mean & range)	FTG after DBS (at 130 Hz)	FTG duration after DBS (sec) (mean & range)	FTG Freq. after DBS (Hz) (5-s window) (mean & range)	notes	EEG channel of greatest coherence
1	4	130	yes	69.8 [35.1–84.9]	74.3 [74–75]	yes	14.3 [9.0–18.8]	74 [73–75]		during R DBS: C3
2	3	130	no	n.a.	n.a.	yes	16.3 [14.3–20.3]	77 [77–77]		after (5 s) R DBS: C3
3	9	4 × 130 3 × 180 2 × 70	yes	59.2 [54.2–66.7]	79.3 [78–81]	yes	38.5 [26.3–57.1]	82 [82–82]	70 Hz: no FTG 130 and 180 Hz: FTG during and after DBS	during R 130 Hz: FCz after R 130 Hz: C4 during R 180 Hz: CP4 after R 180 Hz: Fz
4	5 (long) 12 (short)	long: 3 × 130, 1 × 190, 1 × 180 short: 4 × 70, 4 × 130, 4 × 190	no	n.a.	n.a.	yes	16.5 [7.5–25.6]	81.5 [81–82]	70 Hz: no FTG 130 Hz: FTG after DBS 190 Hz: FTG during and after DBS	after R 130 Hz: CP4 during R 180 + 190 Hz: CP3 after R 190 Hz: CP4
5	4 (long) 5 (short)	130	yes	14.3 [5.8–23.1]	78.5 [78–80]	no	n.a.	n.a.		during L blocks: Fz

### 2.6 Signal processing: pre-processing

All data analysis was performed using custom-written scripts in MATLAB (version 2019a, Mathworks, Massachusetts, USA). Continuous LFP signals were linearly de-trended to remove low-frequency drifts using MATLAB's *detrend* function and DBS artefacts were removed by applying second-order IIR notch filters (Q-factor = 200; see Fig. 1B). These artefacts include line noise at 50 Hz (all recordings), a peak at the subharmonic frequency of 65 Hz during stimulation at 130 Hz in 11/17 STNs (and all 5 patients displaying FTG), and a narrowband signal that appeared at 80 Hz in 8/17 STNs (Fig. 1C; only patients 1 and 2 displaying FTG). The narrowband character of these signals and the fact that they were not delayed in onset and did not outlast the DBS blocks or change in frequency over time differentiated them from the FTG. Spectral amplitudes were estimated between 65 and 90 Hz using the short-time Fast Fourier transform with a window length of 1 s, 25% overlap of consecutive windows and a Hamming window as implemented in MATLAB's *spectrogram* function yielding a frequency resolution of 1 Hz.

### 2.7 Signal processing: FTG detection

We restricted our search for the FTG to the frequency range from 65 to 90 Hz (Kempf et al., 2009; Swann et al., 2016) to avoid DBS subharmonics (only DBS stimulation frequencies of 70, 130, 180 and 190 Hz were used across patients). The presence of FTG was assessed in each STN and stimulation block by visual inspection of both the time-frequency spectrograms and power spectral densities (PSD). To this end, PSDs were computed in three time windows. First, a baseline PSD was computed in a 50-s window before each DBS block (−52 s to −2 s; to avoid contamination from artefacts when DBS was switched on). Second, PSDs were computed during stimulation and finally, post-DBS PSDs were computed in a 5-s window after DBS was switched off. This window length was selected based on visual inspection of the time-frequency maps to include short lasting FTGs persisting after DBS. The PSD from all four epochs was flattened using a fourth order polynomial (*detrend* function) to correct for the 1/f pattern (Pritchard, 1992) and z-scored (Swann et al., 2016). The presence of FTG was determined by the presence of a peak between 65 and 90 Hz that was over two standard deviations above the average of the respective time window using MATLAB's *findpeaks* function (MinPeakHeight: 2) and confirmed by visual inspection of the time-frequency maps. This approach was effective even in 10-s windows if the FTG is present and allows to track the FTG peak during and after DBS (Supplementary Video 1).

### 2.8 Signal processing: FTG amplitude and frequency evolution during DBS

In subjects that showed an FTG in the PSD and time-frequency plot during DBS (see Table 2), the amplitude and frequency profiles of the FTG were further characterised. To this end, we first defined a more constrained region of interest (ROI) in the time-frequency domain. The frequency-axis of the ROI was determined based on the largest PSD peak during the whole DBS block (signal flattened and z-scored as described above) ± 5 Hz. The time-axis equalled the block duration. Further, power estimates within the ROI were binarised. All power estimates that were equal to or above the 70th percentile of all power estimates in the ROI were given the value 1 and all remaining power estimates the value 0. Clusters of connected pixels of value 1 were identified using MATLAB's *bwconncomp* function (8-connected mode; pixels are connected if their edges or corners touch). Note that each estimate corresponds to 1 Hz × 0.75 s (ROI size: 11 Hz x DBS duration in bins of 0.75 s), except in patient 5 where the ROI was changed to 14 Hz x DBS duration*0.25 since the FTG feature was only present in the first quarter of the block and it was further spread along the frequency axis (average ROI size 1666.5 ± 234.1 power estimates). Clusters consisting of less than 15 individual power estimates were removed from the cluster map to exclude possible random fluctuations. All clusters that survived this selection were considered as components of the FTG (see Fig. 3A). Frequencies and amplitudes of the FTG were determined for each time bin (0.75 s, based on settings of the frequency decomposition) for the power estimate with the highest activity within the cluster. To quantify FTG features during and after DBS (Fig. 3, Fig. 4), average FTG frequency and amplitude profiles per subject (mean of all blocks) were z-scored as in:

$${\text{FTG}}_{Z}=\frac{{\mathrm{FTG}}_{S}-\mathrm{mean}\left({\mathrm{FTG}}_{S}\right)}{\mathrm{std}\left({\mathrm{FTG}}_{S}\right)}$$

where FTG_S_ represents the average FTG feature per subject (mean of all blocks) and FTG_Z_ the z-scored FTG feature.

### 2.9 Signal processing: FTG duration, amplitude and frequency evolution after DBS

In subjects that showed an FTG after DBS in the PSD and time-frequency plot, we used a similar method to the above to precisely define the frequency and amplitude profiles of the FTG activity. Differences from the procedure above were as follows. The frequency-axis of the ROI was based on the post-DBS PSD peak and the time-axis was set to 30 s (60 s in subject 3) based on visual inspection (average ROI size: 641.7 ± 67.2 power estimates). Only the largest cluster was kept to define FTG duration, amplitude and frequency. FTG duration was defined as the length of the FTG cluster following cessation of DBS (see Fig. 4A).

### 2.10 Signal processing: LFP-EEG coherence analysis

In all subjects with significant FTG peaks in the power spectrum we computed coherence between the STN-LFP and EEG. EEG signals were linearly de-trended and data from consecutive DBS blocks were concatenated within subjects. Coherence analysis was performed on unfiltered data. Magnitude-squared coherence was computed using the short-time Fast Fourier Transform with window length 0.5 s and 0% overlap as in:

$$\mathrm{Cxy}\left(f\right)=\frac{{\left|P_{\mathrm{xy}}\left(f\right)\right|}^{2}}{P_{\mathrm{xx}}\left(f\right)*P_{\mathrm{yy}}\left(f\right).}$$

where *P*_*xx*_*(f)* and *P*_*yy*_*(f)* correspond to the power spectral densities and *P*_*xy*_*(f)* to the cross power spectral density. Significance levels of coherence were assessed using an upper 95% confidence limit based on the assumption of independence; for details see (Halliday et al., 1995). The EEG channel with greatest coherence per subject is shown in Table 2 and Fig. 7. To compute coherence after DBS blocks, 5-s windows after the last stimulation artefact were concatenated within subjects. EEG-LFP coherence was considered to be present when it exceeded the upper 95% confidence limit at the same frequency as the corresponding FTG in the STN-LFP.

### 2.11 Statistics

Statistical analyses were conducted using custom-written scripts in MATLAB. Comparisons of the FTG features (duration, frequency and amplitude) between the start and end of the stimulation blocks were performed using linear mixed-effects models on individual data. The FTG features were set as dependent variables (raw features) and the first and last 5-s time windows as fixed effects. A similar approach was used to compare the FTG features across consecutive blocks. The normal distribution of each variable and the residuals were visually inspected with quantile-quantile plots and histograms of distribution. All models were estimated by the method of maximum likelihood and included random intercept for subjects to allow different intercepts for each subject, thereby capturing individual differences. Linear mixed-effects models allow for missing data and comparisons of small sample sizes (Muth et al., 2016).

## 3 Results

### 3.1 FTG can be induced by DBS off medication

**Fig. 2:**
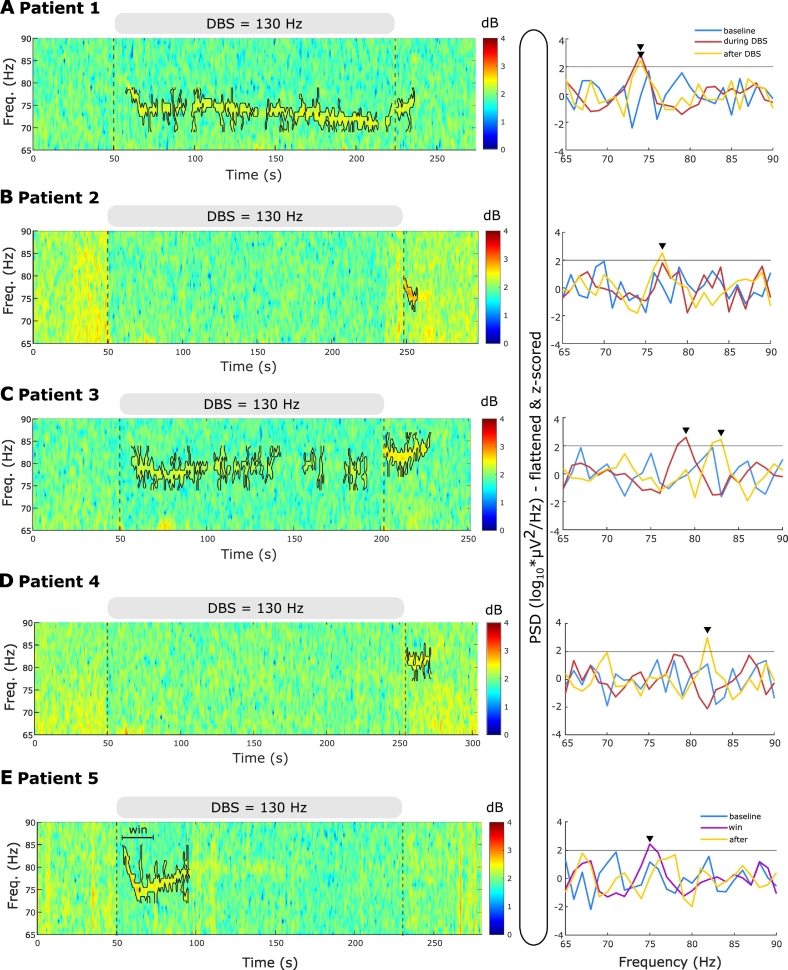
130 Hz DBS induces finely-tuned gamma (FTG) oscillations in the absence of levodopa and dyskinesia. A-E. *Left*: time-frequency spectrograms of representative DBS blocks. The grey bar on top of the trace and the vertical dashed lines denote DBS onset and offset. The FTG feature is surrounded by a contour in each case (based on a 70th percentile threshold of a ROI, see definition in methods section and Fig. 3A). *Right:* Power spectral densities (PSD) during a 50-s baseline period before DBS onset, during the DBS block and during a 5-s window after DBS cessation, with the exception of case 5 (E) in whom the spectrum of a narrower region of interest (win; 20 s long) is presented. The grey horizontal line denotes the FTG threshold at 2 standard deviations above average baseline. Peaks above the threshold are considered significant (black arrowheads). This analysis was confirmed by visual inspection.

Five of 14 patients (35.7%) revealed FTG activity either during or after DBS following overnight withdrawal of antiparkinsonian medication (see Table 1 and significant peaks in PSD of Fig. 2). The FTG arose *de-novo* as it was not discernible during a 50-s baseline period prior to DBS start (Fig. 2). During continuous stimulation at 130 Hz, FTG was observed in the spectrogram of 3 patients (cases 1, 3 and 5) with at least a few seconds delay from stimulation onset (Fig. 2A, C and E). Case 4 displayed an FTG during stimulation at 180 and 190 Hz, and will be considered separately, later.

After DBS, FTG was observed in four patients and changed in pattern. In two patients the FTG outlasted DBS (patient 1 and 3; Fig. 2A+C), whereas in the other two it only started after DBS ceased (patient 2 and 4; see Fig. 2B+D). In 4 of 5 patients that displayed any FTG we observed an additional broad low-gamma rebound after stimulation was stopped (Fig. 1D), as seen in an earlier study (Wiest et al., 2020). This activity spanned from 40 to 70 Hz. Its broadband character and lower frequency range helped to differentiate it from the FTG.

No dyskinesias were reported during or after stimulation in the subjects with FTG (Unified Dyskinesia Rating Scale Part III = 0, (Goetz et al., 2008)), suggesting that FTG can be observed in PD patients off medication without dyskinesia.

### 3.2 FTG dynamics during DBS

**Fig. 3:**
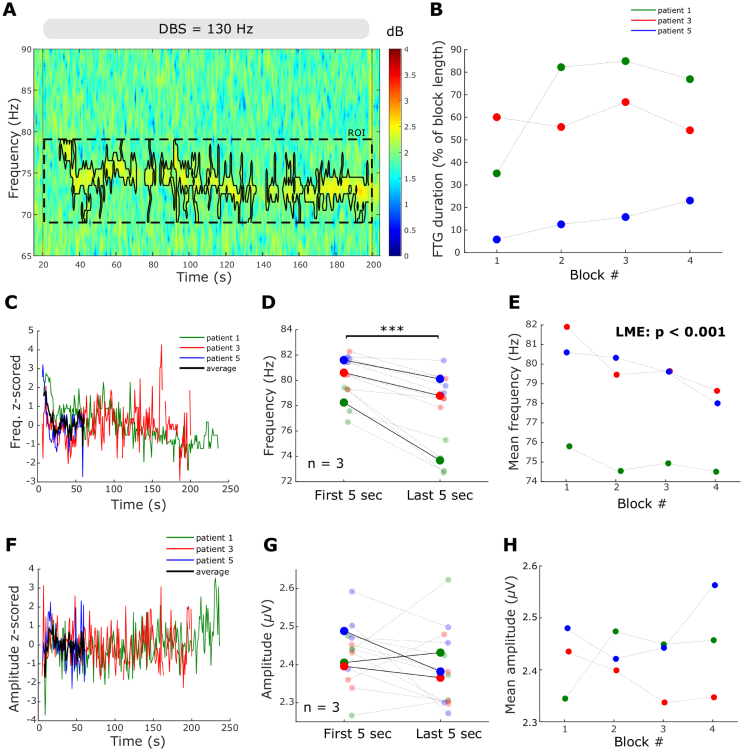
FTG dynamics during DBS blocks. A. Time-frequency spectrogram of a representative DBS block from patient 1. The grey bar and vertical dotted lines denote DBS onset and offset. The black contour surrounds power estimates above the 70th percentile of the ROI (see Methods). B. FTG durations during DBS in percentage of the total length of the stimulation block (linear mixed-effects model (LME): b = 5.89, *t* = 1.85, *p* = 0.09). C. FTG frequency during DBS over time averaged across blocks for each patient (data z-scored to frequencies during DBS; *n* = 3). Note the different FTG durations during DBS. Group average in black only given when data from all 3 patients was available. D. FTG frequency decreases between the first and last 5 s of a DBS block (LME: b = −2.6, *t* = −5.2, *p* < 0.001). Big dots denote averages of patients, small dots denote single blocks. Patients colour coded as in B. E. Mean FTG frequencies decreased across consecutive DBS blocks (LME: b = −0.72, *t* = −4.7, p < 0.001). F. FTG amplitude during DBS over time (data z-scored to amplitudes during DBS; n = 3). G. FTG amplitude in the first and final 5 s of a DBS block (LME: b = −0.02, *t* = −0.7, *p* = 0.47). Big dots denote averages of patients, small dots denote single blocks. H. Average FTG amplitude does not change across consecutive DBS blocks (LME: b = 0.01, *t* = 0.6, *p* = 0.56).

FTG dynamics were further explored during stimulation in patients 1, 3 and 5. As can be observed in Fig. 2, during 130 Hz DBS, the FTG varied between patients. However, in all three the FTG began only after a few seconds of stimulation and demonstrated an initial drop in frequency during the first 20s of stimulation (Fig. 2A, C and E). To better describe the modulation of the FTG in time, we first identified the FTG in the spectral domain using a cluster-based approach (see Methods and Fig. 3A). Subsequently, we extracted duration, frequency and amplitude over time from the identified cluster. On average, the FTG lasted for 77.6 ± 13.5 s (47.8 ± 8.2% of the time during DBS; all blocks of patients 1, 3 and 5 included) with a high inter- and intra-subject variability (see Table 2). The averaged FTG frequency profile suggests a decrease with stimulation duration (Fig. 3C and Supplementary Video 1) which was significant when comparing the average frequency of the first and last 5 s of the FTG in all blocks of all 3 patients (LME: b = −2.6, *t* = −5.2, *p* < 0.001; Fig. 3D). In contrast, there was no significant difference between the initial and final FTG amplitude (LME: b = −0.02, *t* = −0.7, *p* = 0.47; Fig. 3G).

**Supplementary Fig. 1:**
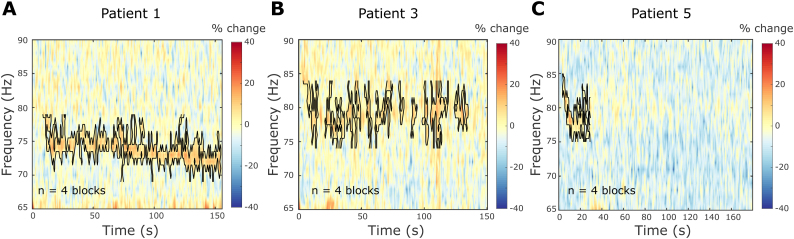
Promotion of FTG during DBS is greater than the promotion of broadband gamma activity. Averaged percentage change of power estimates between 65 and 90 Hz during DBS blocks with respect to a 50-s baseline period prior to DBS start. 4  DBS blocks of slightly different durations were recorded in each patient. Power changes were averaged across blocks and spectrograms are shown for the duration of the shortest block. The FTG feature is surrounded by a black contour (see Methods).

To differentiate the FTG from a broadband gamma increase during DBS, we computed relative changes of power estimates with respect to a 50-s baseline period before DBS onset. Power estimate changes of patients 1, 3 and 5 were averaged across 4  DBS blocks each. The FTG feature showed a greater power increase than adjacent frequencies in the gamma band (Supplementary Fig. 1). Hence, FTG induction is selective and not accompanied by a broadband DBS-induced gamma event-related synchronisation (ERS).

All three patients showing FTG during DBS performed four consecutive blocks at 130 Hz, allowing us to assess FTG dynamics across consecutive DBS blocks. Linear mixed-effects models revealed a significant reduction of the average FTG frequency across DBS blocks (LME: b = −0.72, *t* = −4.7, p < 0.001; Fig. 3E), but no change in average FTG amplitude (LME: b = 0.01, *t* = 0.6, *p* = 0.56; Fig. 3H) or FTG duration during DBS across blocks (LME: b = 5.89, *t* = 1.85, *p* = 0.09; Fig. 3B).

### 3.3 FTG dynamics after DBS

**Fig. 4:**
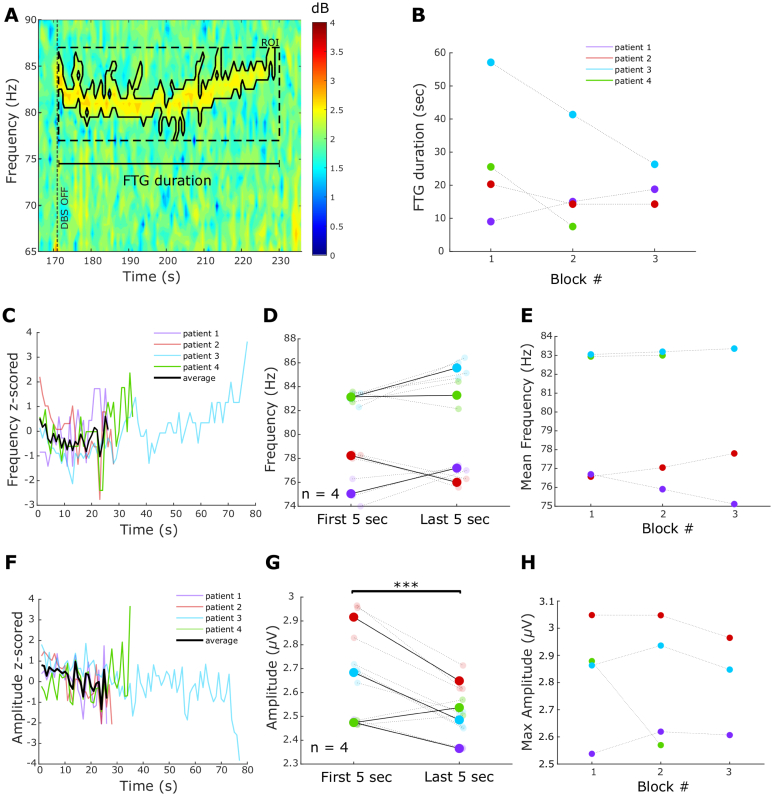
FTG dynamics after DBS blocks A. Representative time-frequency spectrogram of FTG after DBS termination in patient 3. Vertical dashed line denotes the DBS offset and the scale shows FTG duration. The black contour surrounds power estimates above the 70th percentile of the ROI (see Methods). B. FTG duration across blocks. Linear mixed-effects models do not show a significant change (LME: b = −5.26, *t* = −1.59, *p* = 0.15; only first 3 blocks included). C. FTG frequency after DBS over time averaged across blocks for each patient (data z-scored to FTG frequencies after DBS; *n* = 4). Note the different FTG durations after DBS. Group average in black only given when data from all 4 patients was available. D. FTG frequencies after DBS do not differ between the first and last 5 s (LME: b = 0.82, *t* = 1.6, *p* = 0.12). Patients are colour coded as in C. E. Average FTG frequencies across blocks (LME: b = −0.01, *t* = −0.04, *p* = 0.94; only first 3 blocks included). F. FTG amplitude after DBS over time averaged across blocks for each patient (data z-scored to FTG amplitudes after DBS; n = 4). G. FTG amplitude decreases between the first and last 5 s of the post-DBS FTG (LME, b = −0.15, t = −5.2, p < 0.001). H. Maximal FTG amplitudes do not change across consecutive DBS blocks (LME: b = −0.03, *t* = −0.76, *p* = 0.46; only first 3 blocks included).

After discontinuation of 130 Hz DBS, the FTG lasted on average 23.2 ± 4.1 s (Fig. 4C and Table 2). There was no significant difference in frequency between the first and last 5 s (LME: b = 0.82, *t* = 1.6, *p* = 0.12; Fig. 4D). However, post-DBS FTG frequencies appeared to be very variable across patients. While patients 3 and 4 displayed an almost U-shaped frequency curve, FTG frequencies tended to increase in patient 1 and decrease in patient 2. In contrast, the FTG amplitude after DBS decreased (Fig. 4F), with a significant difference when comparing amplitudes between the first and last 5 s (LME: b = −0.15, t = −5.2, p < 0.001; Fig. 4G). Across consecutive blocks, no trends or significant differences were observed in FTG duration (LME: b = −5.26, *t* = −1.59, *p* = 0.15; Fig. 4B), maximal FTG amplitude (LME: b = −0.03, *t* = −0.76, *p* = 0.46; Fig. 4H) and average FTG frequency (LME: b = −0.01, *t* = −0.04, *p* = 0.97; Fig. 4E). Note that only the first 3  DBS blocks were included as it was the minimum number of repetitions performed across the 4 patients showing post-DBS FTG. These results suggest that the FTG appears to be consistent after multiple DBS blocks in individual patients.

### 3.4 FTG frequency does not change with different DBS frequencies off medication

**Fig. 5:**
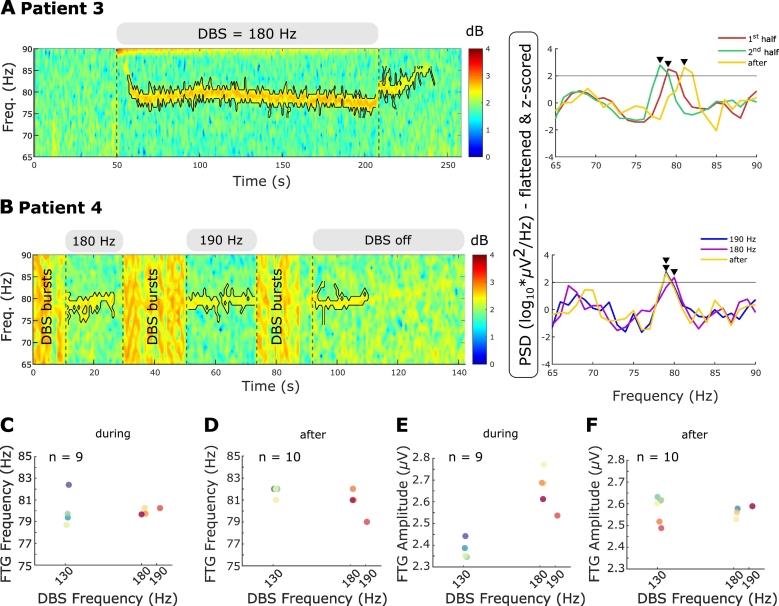
Different DBS frequencies do not change FTG frequency A. *Left:* Time-frequency spectrogram of representative DBS block. Grey bars above the spectrogram and the vertical dashed lines denote DBS onset and offset. Different DBS frequencies are shown in the grey bars. The black contour surrounds the FTG feature within the ROI (see Methods). PSD shown for the first and second half during DBS and the 5-s reference period after DBS cessation. Grey horizontal line denote the FTG threshold at two standard deviations above average of the respective reference periods. Black arrowheads denote significant PSD peaks. B. *Left:* Time-frequency spectrogram of DBS at 180 and 190 Hz. Both DBS blocks are separated by DBS bursts (label: DBS bursts) consisting of repetitive periods on and off DBS of variable length. *Right:* PSD shown for periods during DBS at 180 and 190 Hz and a 5-s reference after DBS ceased. Horizontal line, contours and arrowheads as in A. C + D. FTG frequencies during (C) and after (D) long DBS blocks plotted over DBS frequencies at 130 Hz, 180 Hz and 190 Hz (all blocks from patients 3 and 4). Despite varying DBS frequencies, the FTG stays at a similar frequency. E + F. FTG amplitudes during (E) and after (F) long DBS blocks plotted over DBS frequencies at 130 Hz, 180 Hz and 190 Hz (all blocks from patients 3 and 4). During DBS, FTG amplitudes tend to be increased at higher driving frequencies.

**Fig. 6:**
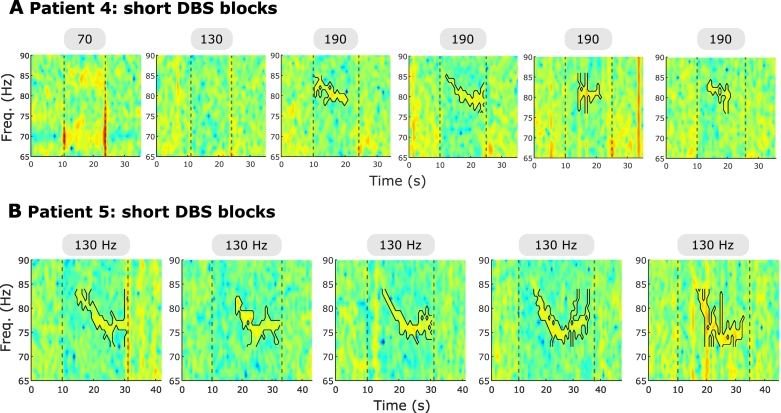
FTG in short DBS blocks. A. In patient 4, short DBS blocks of on average 13.1 ± 0.2 s (separated by resting periods of 149.0 ± 3.9 s) were tested at different DBS frequencies. The grey bars and vertical lines denote DBS onset and offset. DBS frequencies are shown in the boxes. Every frequency was tested four times (only one block shown for 70 and 130 Hz). Note the notch filter at 70 Hz when stimulation was at 70 Hz. Colour bar as in Fig. 6A. The FTG feature is surrounded by a contour where present. B. In patient 5, short DBS blocks of on average 22.4 ± 1.2 s (separated by resting periods of 177.2 ± 18.6 s) were tested at 130 Hz. The grey bars and vertical lines denote DBS onset and offset. Colour bar as in Fig. 6A. The FTG feature is surrounded by a contour.

In two patients that displayed FTG during or after 130 Hz DBS, we tested if different DBS frequencies elicited changes in FTG peak frequency, suggestive of entrainment. In patient 3, DBS was tested at 70 Hz and 180 Hz, as well as the standard 130 Hz. Stimulation at 70 Hz did not induce an FTG, however, we cannot preclude the presence of an FTG feature underneath the stimulation artefact or its subharmonics. Stimulation at 180 Hz led to an FTG at about 80 Hz, similar to the FTG elicited by DBS at 130 Hz (compare Fig. 5A with Fig. 2C and Supplementary Video 1). In contrast to the artefact at 80 Hz (Fig. 1C), this 80 Hz-signal started with a delay after DBS onset and outlasted stimulation. Moreover, it showed dynamic changes and was hence considered as an FTG feature. In patient 4, DBS was delivered at 180 Hz and at 190 Hz separated by short stimulation bursts consisting of repetitive periods on and off DBS at these frequencies (Fig. 5B). While DBS at 130 Hz only elicited an FTG after DBS in this patient (Fig. 2D), DBS at 180 Hz and 190 Hz induced a significant FTG peak during stimulation which outlasted DBS. FTG frequency was at about 80 Hz for all three DBS frequencies (compare Fig. 5B and Fig. 2D). While the patients and block numbers are too small to make definitive inferences, two trends are apparent: Firstly, FTG frequencies remained reasonably constant during and after DBS at different driving frequencies (Fig. 5C+D) and, secondly, FTG amplitudes during DBS seemed to increase with DBS frequency (Fig. 5E) while FTG amplitude after DBS was similar at 130, 180 and 190 Hz stimulation (Fig. 5F). The latter is underpinned by the results observed in short blocks of DBS (four blocks per DBS frequency of on average 13.1 ± 0.2 s) at 70 Hz, 130 Hz and 190 Hz in patient 4. While DBS at 70 Hz and 130 Hz did not induce an FTG meeting our criteria either during or after DBS, 190 Hz stimulation triggered an FTG during short blocks (Fig. 6A). In patient 5, the FTG was evident in short blocks of on average 22.4 ± 1.2 s even at 130 Hz (Fig. 6B) with a similar profile as in long blocks but cut short by DBS termination.

### 3.5 LFP-EEG coherence at FTG frequencies

**Fig. 7:**
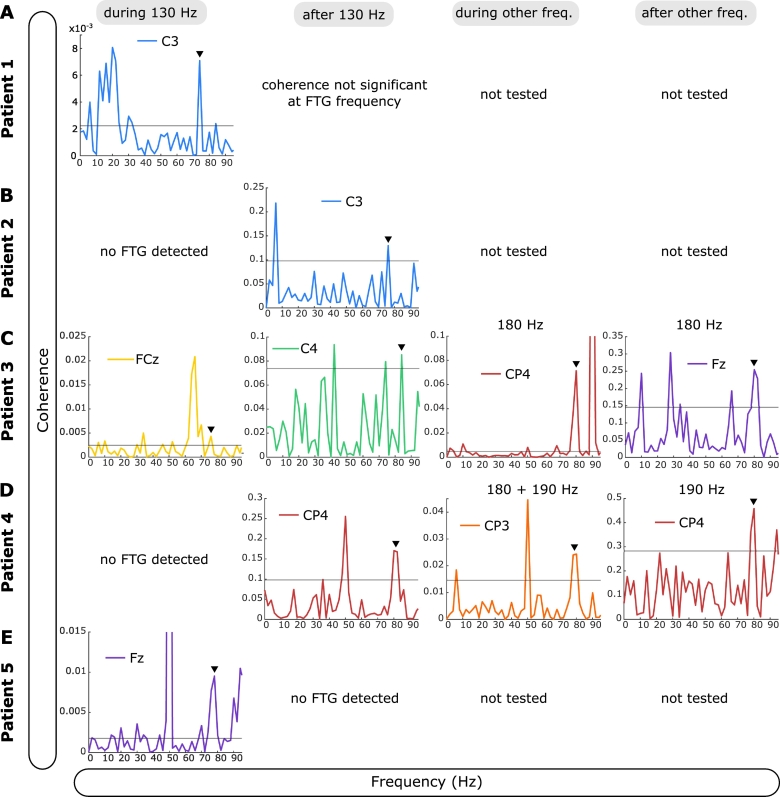
LFP-EEG coherence at FTG frequencies. A.-E. EEG channel with greatest coherence is shown for all FTG subjects. For during blocks analysis, time series were concatenated within subjects. For after block analysis, signals in a 5-s time window following cessation of DBS were concatenated within subjects. In patient 4 and 5 short and long DBS blocks were concatenated together. If not 130 Hz, DBS frequencies are shown on top of the plots. EEG channels are colour coded. Black horizontal line denotes the significance level (see Methods). Black arrowheads indicate the corresponding frequency of the FTG peak in the STN-LFP power spectrum. Coherence at FTG frequency was significant in all subjects shown. Note that artefactual coherence is variously seen at 50 Hz (line noise), 65 Hz (subharmonic of 130 Hz DBS) and 90 Hz (subharmonic of 180 Hz DBS).

Since the FTG has previously been detected in cortical recordings we studied LFP-EEG coherence. We found significant LFP-EEG coherence at FTG frequencies in all the patients exhibiting FTG in their STN-LFP (Fig. 7 and Table 2). Based on patients 3 and 4, coherence tended to be greater at higher DBS frequencies (Fig. 7C+D) similar to the increased FTG amplitude during blocks of increased DBS frequencies.

## 4 Discussion

In this study, we show that STN-DBS induces FTG in the STN-LFP of about a third of patients after overnight withdrawal of antiparkinsonian medication. As previously reported, we also found significant coherence at FTG frequencies between the STN-LFP and EEG. However, unlike the FTG described in PD patients on dopaminergic medication, we recorded FTG while dyskinesias were absent. Furthermore, there was no evidence to suggest a shifting of the FTG frequency in the STN according to stimulation frequency off medication, and FTG could be maintained for ~20 s after DBS. These observations suggest that off medication the FTG was not entrained by DBS, although we did find that FTG frequencies subtly, but significantly, declined within and across consecutive DBS blocks.

### 4.1 Was FTG induced or entrained by DBS?

Swann et al. (2016 and 2018) previously demonstrated that the FTG recorded at the cortical level in PD patients on medication may be entrained by high frequency stimulation of the STN when patients experience dyskinesia. This entrainment was manifest as a shift in the frequency of the FTG peak to that of the subharmonic of the stimulation frequency. Another study reported a correlation between power at FTG frequencies in STN and cortex and local power at the stimulation frequency (Muthuraman et al., 2020). However, this study could not establish entrainment through a shifting of FTG frequency to match subharmonics of the stimulation frequency, and did not directly demonstrate endogenous FTG at the stimulation sites during stimulation in the off medication state. In the current study, also off medication, we were able to record *de-novo* FTG, and demonstrate that it outlasted stimulation by on average 20s, or even appeared for the first time during this period. These relatively sustained stimulation after-effects in addition to the stability of FTG frequencies independent of the DBS frequency argue against simple entrainment of the FTG by DBS.

The lack of evidence of shifts in FTG frequency (or in LFP-EEG coherence) with different stimulation frequencies would also be against direct entrainment of FTG. Here, however, some caution is required. We recorded both a stereotyped frequency FTG in the power spectra density during stimulation, and signal at stimulation subharmonics. Totally discounting a physiological contribution to those subharmonics is difficult, although their narrow, fixed frequency and their duration precisely matching the duration of stimulation might point more to stimulus artefact. Also of note, the LFP-EEG coherence during DBS only disclosed a peak at the same frequency as the FTG in corresponding power spectra of the LFP and not at half the stimulation frequency in patients 1, 4 and 5 (Fig. 7).

In summary, the evidence argues against entrainment of the FTG by DBS off medication and is more in favour of the inducing of FTG as stimulation reconfigures networks to support this oscillation, perhaps paralleling how levodopa reconfigures networks to induce FTG (Jenkinson et al., 2013). Interestingly, the duration over which FTG outlasted stimulation is similar to the time beta oscillations take to return to pre-stimulation levels (Wiest et al., 2020), and corresponds to the delayed recovery of bradykinesia after cessation of DBS (Kühn et al., 2009).

Why might STN-DBS entrain FTG when patients are dyskinetic on medication in the case of the study by Swann et al. (2016), but not when not dyskinetic off medication, as here? The former study sampled FTG from sensorimotor cortex, but this is unlikely to be responsible for the difference given that STN and cortical activities are coherent at FTG frequencies as reported previously (Brown et al., 2001; Cassidy et al., 2002; Litvak et al., 2012; Williams et al., 2002) and confirmed here. Perhaps more likely the difference arises from the effects of dopaminergic therapy. When this medication is withdrawn overnight, spontaneous FTG in the STN is absent or diminished (Jenkinson et al., 2013), and when induced by stimulation FTG does not shift in frequency according to the frequency of stimulation. On dopaminergic medication FTG is exaggerated and sharply tuned (in those recordings displaying such activity), suggesting an under-damped oscillation that is more likely to be subject to resonance-related phenomena like amplitude enhancement and entrainment by driving frequencies, or subharmonics thereof, that are relatively closely related in frequency. In summary, although off medication DBS may induce, but not entrain, FTG, the situation may be different on medication.

### 4.2 Why is the FTG an inconsistent finding across patients?

Only about a third of patients had an FTG induced by stimulation. One explanation might be that the FTG was obscured by a broadband power suppression during DBS in many patients. However, this is unlikely given that power suppression is only significant in the beta and low-gamma range (Wiest et al., 2020). Another explanation might be that patients without FTG were under-stimulated. However, stimulation amplitudes ranged from 1.8–4 mA in both FTG and non-FTG subjects (Table 1). Instead, it is possible that the factors determining the presence or absence of FTG during or immediately after stimulation might be similar to those determining the presence or absence of FTG in patients on medication, where a similar proportion of patients display FTG (López-Azcárate et al., 2010). While the FTG can be detected in a widely distributed network, including thalamus, internal segment of the globus pallidus, STN and cortex, within these sites it may be highly focal. In the STN, in particular, FTG arises within the dorsolateral STN and adjacent Zona incerta (Lofredi et al., 2018; Trottenberg et al., 2006), raising the possibility that minor differences in electrode placement may account for the presence or absence of FTG within our, and other, cohorts. These differences, if they exist, were too small to be picked up by the imaging co-registration techniques in our limited sample (see Table 1). It could also be that the processes supporting gamma synchrony are susceptible to the stun effect of surgery, but chronic recordings in the same patients will be necessary to establish this. Finally, there might be phenotypic differences that determine the presence of FTG. Of these, the propensity for dyskinesias has received the most interest (Alonso-Frech et al., 2006; Fogelson et al., 2005; Swann et al., 2016). The induced FTG lasted 10s of seconds following the cessation of stimulation which mirrors the time necessary for DBS-induced dyskinesias to subside. However, we studied patients in the off medication state, without dyskinesias. The FTG therefore does not seem to be exclusively linked to dyskinesias (see Table 1) as it has also been shown in invasive recordings from patients suffering from dystonia and myoclonus epilepsy at rest (Kempf et al., 2009). At best therefore, the FTG induced by stimulation in the off medication state may be a trait marker of the propensity for dyskinesias, even if on medication it may be more directly involved in the generation of these involuntary movements (Swann et al., 2016; Swann et al., 2018).

### 4.3 Study limitations

Our study has a relatively low sample size of 14 patients (17 STNs), with only a minority of these showing an induced FTG. Although we used linear mixed-effects models which allow for comparisons of small sample sizes (Muth et al., 2016), we acknowledge that our results should be interpreted carefully and require corroboration in a larger sample. In addition, recordings shortly after DBS surgery may be confounded by transient symptom alleviation and circuit disruption due to electrode implantation (Chen et al., 2006; Mann et al., 2009).

## 5 Conclusions

We report FTG oscillations that arise *de-novo* during or immediately after STN-DBS in the off-medication state, which can be seen in the STN-LFP and in the coherence between this and EEG. The response to different stimulation frequencies and the prolonged stimulation after-effects suggest that in the off medication state the FTG is induced rather than entrained, providing evidence of a stimulation-related reconfiguration of basal ganglia circuits to support gamma oscillations. The presence of slow changes in FTG frequency within and across consecutive DBS blocks suggests that such circuit reconfiguration may itself subtly evolve over time.

The following are the supplementary data related to this article.

Supplementary Video 1 FTG dynamics during and after DBS. *Left:* Time-frequency spectrogram of a 180 Hz DBS blocks (see Fig. 6A). Dotted vertical lines denote onset and offset of stimulation. Moving black box contains a 10-s window which was used to compute the power spectrum on the right. *Right:* Flattened and z-scored power spectrum of the respective reference box from the left. Black horizontal line denotes the significance level at 2 standard deviations above the mean. Black arrowhead tracks the significant FTG peak. Supplementary Video 1

## Credit author statement

Christoph Wiest: Writing – Original Draft, Investigation, Formal analysis, Visualization

Gerd Tinkhauser: Funding acquisition, Investigation, Writing – Review & Editing

Alek Pogosyan: Software

Shenghong He: Software

Fahd Baig: Investigation

Francesca Morgante: Investigation

Abteen Mostofi: Investigation

Erlick A. Pereira: Resources

Huiling Tan: Writing – Review & Editing, Funding acquisition

Peter Brown: Funding acquisition, Project administration, Conceptualization, Resources, Writing – Original Draft, Supervision

Flavie Torrecillos: Conceptualization, Investigation, Formal analysis, Validation, Writing – Review & Editing, Supervision, Project administration

All authors approved the manuscript before submission.

## Declaration of Competing Interest

The authors declare no competing financial interests.
